# An Unsupervised Transfer Learning Framework for Visible-Thermal Pedestrian Detection

**DOI:** 10.3390/s22124416

**Published:** 2022-06-10

**Authors:** Chengjin Lyu, Patrick Heyer, Bart Goossens, Wilfried Philips

**Affiliations:** TELIN-IPI, Ghent University-imec, St-Pietersnieuwstraat 41, B-9000 Ghent, Belgium; patrick.heyerwollenberg@ugent.be (P.H.); bart.goossens@ugent.be (B.G.); wilfried.philips@ugent.be (W.P.)

**Keywords:** pedestrian detection, unsupervised transfer learning, domain adaptation, deep learning, multispectral fusion

## Abstract

Dual cameras with visible-thermal multispectral pairs provide both visual and thermal appearance, thereby enabling detecting pedestrians around the clock in various conditions and applications, including autonomous driving and intelligent transportation systems. However, due to the greatly varying real-world scenarios, the performance of a detector trained on a source dataset might change dramatically when evaluated on another dataset. A large amount of training data is often necessary to guarantee the detection performance in a new scenario. Typically, human annotators need to conduct the data labeling work, which is time-consuming, labor-intensive and unscalable. To overcome the problem, we propose a novel unsupervised transfer learning framework for multispectral pedestrian detection, which adapts a multispectral pedestrian detector to the target domain based on pseudo training labels. In particular, auxiliary detectors are utilized and different label fusion strategies are introduced according to the estimated environmental illumination level. Intermediate domain images are generated by translating the source images to mimic the target ones, acting as a better starting point for the parameter update of the pedestrian detector. The experimental results on the KAIST and FLIR ADAS datasets demonstrate that the proposed method achieves new state-of-the-art performance without any manual training annotations on the target data.

## 1. Introduction

**Figure 1:**
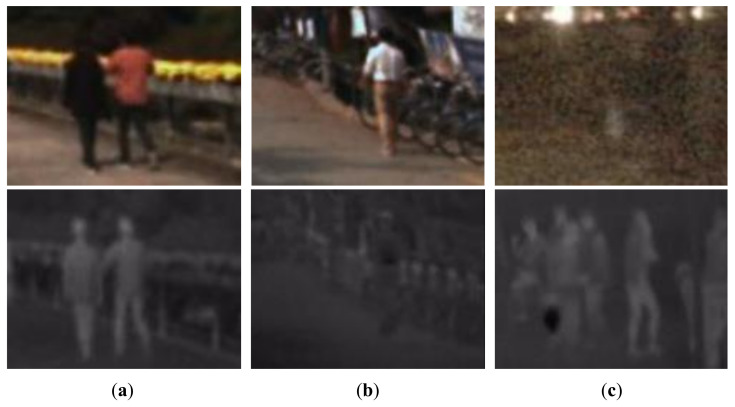
Typical samples of multispectral pedestrian images. The first and second row are visible and thermal images, respectively. (**a**) Both visible and thermal images are captured in good conditions. (**b**) Thermal image is captured at ambient temperatures similar to the body temperature and the thermal crossover may occur, leading to blurred and low-contrast results, while the quality of visible image is still good. (**c**) Visible image is captured under an extreme lighting condition, while their paired thermal image is robust against the illumination change.

As one of the essential tasks in the field of computer vision, pedestrian detection has been widely discussed and investigated over the past decades. Pedestrian detection plays a crucial role in various applications, such as autonomous driving [1,2], public surveillance [3,4], care for the elderly [5], and intelligent transportation systems [6,7]. Most of the successful pedestrian detectors are restricted to the images sensed by visible cameras with a good lighting condition and might fail to work when there is insufficient illumination, e.g., during the night time or adverse weather conditions. Different from the conventional cameras that sense images using visible light, thermal cameras operating in the infrared spectrum could capture the infrared radiation reflecting the temperatures of a sensed object and its background environment. Regarding pedestrian detection, a pedestrian usually has a distinct thermal signature, including the shape and temperature features compared to the background being viewed. It is robust to detect pedestrians from a thermal camera against illumination changes. Nevertheless, thermal cameras also have some shortcomings for pedestrian detection, such as sensing fewer detailed appearance features and background textures than visible cameras. Additionally, there is the so-called thermal crossover phenomenon where pedestrians might get indistinguishable from the background environment when their temperatures are similar [8]. Some typical examples could be found in Figure 1.

To tackle these issues, researchers have proposed solutions for pedestrian detection based on dual-camera systems with a visible and thermal camera pair [9]. Generally speaking, the visible and thermal cameras could provide complementary information compared to a single-modality camera to improve the performance of pedestrian detection. Images captured by a visible camera can provide detailed visual appearance details of pedestrians depending on good illumination. In contrast, the thermal camera is not sensitive to the surrounding illumination condition and could provide robust human silhouettes. Thus, it is helpful to combine the advantages of both cameras and fuse the complementary characteristics from the two separate modalities to achieve robust pedestrian detection under challenging illumination and weather conditions.

The majority of current research focuses on designing an appropriate fusion strategy to exploit the multispectral information from the dual cameras [10,11,12]. To the best of our knowledge, although these methods that implement fusion detectors based on DCNNs have obtained remarkably good performance, the current pedestrian detectors may be biased towards the popular benchmarks in the single-dataset training and test pipeline, thus reducing their generalization capability [13]. The real-world data contain images captured under various illumination and weather conditions, making the direct evaluation performance (i.e., without retraining the detector) not optimal [14]. Usually, to guarantee the performance of a pedestrian detector in a new scenario, human annotators need to label the new data for the supervised training process. A qualified annotator is required to be an expert at distinguish pedestrians on both visible and infrared spectra for the labeling of multispectral images. Moreover, the labeling work is labor-intensive and time-consuming, making the deployment of a pedestrian detector unscalable. For that reason, it is beneficial to design a method that could adapt detectors from the source dataset to the target domain.

Here, we define unsupervised transfer learning as the case of having abundant labeled source data and no labeled target training data. As far as we know, only limited initial work [15,16] exists on unsupervised transfer learning in the area of visible-thermal pedestrian detection. Inspired by their idea of using pseudo training labels, we proposed a basic unsupervised transfer learning framework in our prior paper [17] to adapt pedestrian detectors to new scenarios, where the pseudo labels are generated to update the parameters of a detector.

**Figure 2:**
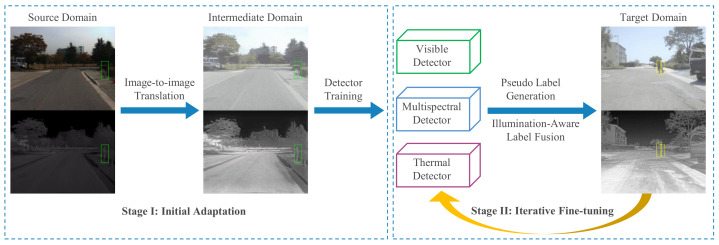
Illustration of the proposed scheme. The overall process consists of two stages: initial adaptation and iterative fine-tuning. The initial adaptation stage aims at handling the domain shift problem across the source and target domains. The iterative fine-tuning stage is adopted to converge the multispectral pedestrian detector on the target domain progressively, based on illumination-aware fused pseudo labels.

In this paper, we extend the prior work into a novel and unified framework. The overall framework consists of two key steps: initial adaptation and iterative fine-tuning (as shown in Figure 2). Since there is usually an obvious domain gap between the source and target domains, we construct an intermediate domain lying between the source and target multispectral data by contrastive learning, to reduce the domain gap. We perform the initial adaptation by training the detector on the intermediate domain. After that, initial pseudo labels of high quality are generated for the subsequent fine-tuning of the detectors. Given that the environmental illumination has different impacts on the detection results from different modalities [18], the use of estimated illumination level is investigated to guide the pseudo label fusion in our work. By conducting extensive experiments, the effectiveness of the proposed framework is validated. The main contributions are as follows:

- A novel and unified unsupervised transfer learning framework for multispectral pedestrian detection is proposed. The adaptation of a multispectral detector using pseudo training labels, leveraging auxiliary detectors specializing in single modalities;
- The idea of using an intermediate domain representation is introduced to reduce the domain gap between the source and target domains. The high-quality initial pseudo labels are generated based on the this intermediate domain;
- An illumination-aware label fusion strategy is proposed to select the best pseudo labels from different modalities, where the environmental illumination level is accessed by an independent illumination estimation network;
- Experimental results on the KAIST [9] and FLIR ADAS [12] datasets demonstrate that our proposed method outperforms the state-of-the-art unsupervised method and reports comparable performance with supervised training.

The rest of this paper is organized as follows. Section 2 reviews the related work focused on pedestrian detection in the literature. Section 3 introduces the proposed framework and its implementation details. Next, Section 4 demonstrates the experimental results and discussion. Finally, the conclusion of this paper and potential future work directions are presented in Section 5.

## 2. Related Work

### 2.1. Visible Pedestrian Detection

Pedestrian detection is one of the most fundamental tasks in many computer vision applications. In recent years, many methods have been proposed for pedestrian detection, and most of these research works are based on visible cameras due to the high resolution and low price. Contemporary pedestrian detection methods have developed rapidly from handcrafted features to deep learning approaches. In the year 2005, Dalal and Triggs [19] proposed the histograms of oriented gradient (HOG) descriptors for pedestrian detection. Later, inspired by the HOG descriptor, Felzenszwalb et al. [20] proposed the deformable part models (DPM), which can describe a human as a collection of different parts to improve the robustness of the method. Dollár et al. [21] investigated the integral channel features (ICF) on pedestrian detection. Further, they proposed a variant of ICF called aggregated channel features (ACF) [22], which is one of the most successful handcrafted feature-based pedestrian detection approaches.

With the advent of DCNNs, methods based on deep learning have predominated the research on pedestrian detection. Generally, these methods fall into two main categories: two-stage and single-stage detectors. The region-based convolutional neural network (R-CNN) [23] initiated a two-stage work using selective search to generate region proposals for detection. Based on the R-CNN framework, various solutions have been proposed to speed up the detection, such as Fast R-CNN [24] and Faster R-CNN [25]. Faster R-CNN realizes the end-to-end detection relying on pure DCNNs to perform both region proposal and classification, which makes it a standard and popular baseline for pedestrian detection [26]. Derived from the Faster R-CNN architecture, a variety of pedestrian detection methods have been introduced [27,28,29]. On the other hand, the most widely used one-stage detectors belong to the YOLO [30], and SSD [31] families. Compared to the two-stage detectors, those one-stage detection frameworks can directly predict the output bounding boxes without the intermediate region proposal process, leading to a simpler and faster model architecture while losing the accuracy performance sometimes.

### 2.2. Thermal Pedestrian Detection

Although pedestrian detection based on visible cameras has been widely used in many applications, satisfactory performance cannot be guaranteed at nighttime or under low lighting conditions. Recently, pedestrian detection on thermal imagery has attracted increasing attention due to its superiority in low-illumination conditions. In practice, most of the current research benefits from the excellent works on visible imagery, such as the popular HOG descriptor and Faster R-CNN framework. Chang et al. [32] proposed an early work based on the HOG descriptor, where AdaBoost is used to perform the detection task. In [33], the fusion of HOG features and local binary patterns in thermal modality showed an impressive performance in the age of handcrafted features.

Similar to the development of visible pedestrian detectors, the success of DCNNs has advanced the detection based on thermal data significantly. However, the natural characteristics of thermal images, such as low resolution and blurred details, restrict the further improvement of the performance. Ghose et al. [34] adopted saliency maps to augment the thermal images and thus improve the training of Faster R-CNN in thermal images. In [35,36], the authors presented enhancement methods based on generative adversarial network (GAN) for thermal pedestrian detection. Moreover, the work of [35] borrowed visible training data from other sources to realize enhancement for the so-called pseudo multispectral pedestrian detection as a compromise for the absence of paired multispectral data, which proves the importance of real visible-thermal multispectral data and pedestrian detection based on it.

### 2.3. Multispectral Pedestrian Detection

For a pedestrian detection task, the fusion of sensors from multiple modalities (e.g., cameras, LiDAR, and radar) can provide more useful information to achieve robust performance for self-driving and public video surveillance applications. Among all these sensors, a visible-thermal multispectral camera is one of the most common choices due to the vision-like sensory for human intervention, and lower price compared to other fusion solutions [37]. Like many modern computer vision tasks, the research on visible-thermal multispectral pedestrian detection is also data-driven. The quality of datasets predetermines the performance of pedestrian detectors, especially those based on DCNNs. The recently published large-scale datasets, such as KAIST [9], CVC-14 [38] and FLIR ADAS [12], have attracted much research attention on the fusion of multispectral information for all-day pedestrian detection applications.

Hwang et al. [9] introduced a multispectral ACF encoded to include additional HOG features of the thermal images. Liu et al. [10] first introduced Faster R-CNN into this area and proposed four typical architectures where the fusion is implemented at different stages. Among all these four methods, Halfway Fusion achieves the best performance. From then on, Faster R-CNN has been adopted as a standard baseline in this area by many researchers because it is fair and clear to determine whether a tested improvement is due to the proposed fusion method or the detector itself. König et al. [11] utilized boosted decision trees (BDT) instead of the original classification network in Faster R-CNN to reduce potential false positives. Recently, to facilitate more fine-grained fusion, sorts of solutions have been proposed with the help of semantic information [39,40], attention mechanisms [41,42] and illumination-aware weighting [18,43]. Specifically, there are two types of illumination-aware weighting designs: built into the detector [43] or independent illumination network [18], depending on whether it is built on the computed features in the Faster R-CNN detector. Meanwhile, there are also several recent works focusing on real-time multispectral pedestrian detection based on one-stage frameworks such as YOLO and SSD [44,45,46]. Recently, Li et al. [46] proposed a method integrating both feature-level fusion and decision-level fusion to ensure reliable detection.

### 2.4. Unsupervised Transfer Learning

Many works have been proposed for unsupervised transfer learning in the context of image classification [47,48], while the object detection task consisting of both localization and classification is much more complicated. This brings unique challenges and has attracted growing attention very recently. A pioneering work for object detection belongs to Chen et al. [49], where the problem of domain shift was addressed on both image-level and instance-level by an adversarial training manner. Saito el al. [50] proposed a method to focus on the adversarial alignment of local similar features. Hsu et al. [51] proposed to bridge the large gap between domains with an intermediate domain, with the help of an image-to-image translation network CycleGAN [52]. Recently, Zhang et al. [53] presented a coarse-to-fine adaptation method to minimize the feature distance between the same object category from different domains.

Although multispectral pedestrian detectors trained on a specific dataset have achieved superior performance, the generalization ability across datasets is limited, leading to retraining the detector based on new training data [14]. Multispectral data captured from different real locations have the domain shift problem, as the visible cameras are sensitive to the illumination conditions and the thermal images have different appearances according to the environment temperature ranges. To retain the optimal detection performance, the newly collected data demand the annotation work from experienced experts for retraining, which makes the real-world deployment slow and unscalable. Thus, unsupervised transfer learning is utilized to adapt the detectors to the unlabeled target domain, leveraging the knowledge from the source domain [54].

As far as we know, there is limited unsupervised transfer learning work in the area of multispectral pedestrian detection. Cao et al. [15] proposed an unsupervised approach to adapt a generic pedestrian detector to the target multispectral domain without using any target annotations. Later, Guan et al. [16] combined the pedestrian detection with semantic segmentation and utilized pseudo annotations to adapt the multispectral detector iteratively. The final output of their designed detector [16] is in the form of full-size heat maps instead of bounding boxes. Both of these two methods are implemented based on pseudo labels, while the large domain gap and varying illumination conditions influence the quality of generated pseudo labels. How to alleviate these challenging problems has not been studied yet.

## 3. Methodology

This section introduces the proposed framework to perform unsupervised transfer learning (UTL) for multispectral pedestrian detection. Firstly, we present an overview of the whole framework. Then, the intermediate domain is presented as a bridge between the source and target domains. Finally, the proposed illumination-aware label fusion strategy is demonstrated.

### 3.1. Framework Overview

**Figure 3:**
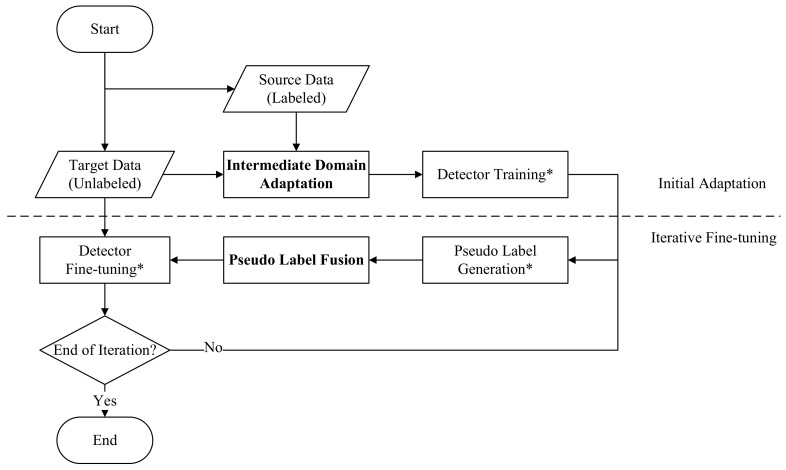
The flow chart of our proposed framework. The main unsupervised adaptation process is divided into two steps: initial adaptation and iterative fine-tuning. In the first stage, the intermediate visible and thermal images are generated by mimicking the target domain style. The initial adaptation is performed by training a detector using the intermediate data with source labels. In the second stage, pseudo labels of the target data generated by both multispectral and auxiliary single-modality (i.e., visible and thermal) detectors, are fused via an illumination-aware mechanism. The iterative fine-tuning process is adopted to converge the detector on the target domain progressively. A block marked with “*” means that the operation is performed for both multispectral and auxiliary detectors.

The task of our proposed UTL multispectral pedestrian detection framework is to adapt a multispectral pedestrian detector from the source domain S consisting of the source visible-thermal image pairs $\left\{X_{s}^{V};X_{s}^{T}\right\}$ and manual annotations $Y_{s}$ to the target domain T with only data $\left\{X_{t}^{V};X_{t}^{T}\right\}$, where *V* represents the visible spectrum and *T* is the thermal modality respectively. The overall framework consists of two main stages, i.e., initial adaptation and iterative fine-tuning. An overview of the proposed framework is presented in Figure 3.

Since there are common domain shift problems across datasets for both visible and thermal modalities, we firstly construct the intermediate domain M lying between the source and target domains, to perform the initial adaptation. An image-to-image translation technique is applied to the source visible-thermal image pairs to generate the corresponding intermediate image pairs $\left\{X_{m}^{V};X_{m}^{T}\right\}$ in the intermediate domain M, which matches the global domain style of the target while keeping the local image content. Consequently, this synthetic domain located between the source and target domains can help reduce the large domain gap between S and T. Later, based on the generated intermediate images $\left\{X_{m}^{V};X_{m}^{T}\right\}$ and the source training labels $Y_{s}$, the initial adaptation S→M is finished. In this way, an initially adapted detector benefits from knowledge from both the source and target domains. A detailed description of the intermediate domain adaptation can be found in Section 3.2.

After that, the initially adapted multispectral detector goes through a second-stage fine-tuning process M→T based on iteratively generated training pseudo labels in the target domain. When the environmental illumination level is high (e.g., during the sunny daytime), a visible pedestrian detector generates good detection results, while a thermal pedestrian detector still works for a low-illumination detection scenario. Considering that these two single-modality detectors output reliable results under certain conditions, we adopt them as auxiliary detectors for the pseudo label generation. An illumination-aware label fusion strategy is proposed to fuse the generated pseudo labels. In particular, a tiny illumination estimation network (IEN) is introduced for label fusion, as illustrated in Section 3.3.

### 3.2. Intermediate Domain Adaptation

As the data distribution varies across datasets, the unsupervised adaptation of a pedestrian detector between two distant domains is a considerably challenging task. For instance, the sensed ambient temperature ranges can influence the thermal imaging results dramatically: a human body is brighter than the background on cold days while being darker in hot days. Meanwhile, the image contrast also varies according to the sensed temperature ranges. As for the visible images, they are relatively sensitive to the lighting conditions. To achieve the domain adaptation for object detection in the color imagery, Hsu et al. [51] proposed to bridge the large domain gap between the source and target domains with an intermediate domain, with the help of an image-to-image translation network CycleGAN [52]. Inspired by this work, we introduce the idea of intermediate domain into the task of multispectral pedestrian detection, to generate intermediate visible and thermal images, making the adaptation easier.

Given a set of image $\left\{X_{s}^{V};X_{s}^{T}\right\}$ from the source domain S, the intermediate images $\left\{X_{m}^{V};X_{m}^{T}\right\}$ of the intermediate domain M are generated via image style translation to match the appearance style of the target domain T, while retaining the local content of interest. In pedestrian detection, an ideal intermediate domain should imitate the domain style of the target, e.g., illumination and weather conditions. This kind of domain style can be learned via an adversarial loss [55]. Meanwhile, the locations and sizes of pedestrians, which are the vital local contents from the source, should not be changed. In general, a good domain translation method for pedestrian detection is supposed to have the ability to keep the local image content from the source data while generating a domain style globally similar to the target domain.

To achieve this goal, a contrastive learning-based domain style translation method called CUT [56] is employed in this paper. Different from the work based on cycle-consistency [52], which changes the local image content together with the global style, the CUT method presents a straightforward yet efficient way of maintaining correspondence in local content for global domain style translation by maximizing the mutual information between the source and generated intermediate domains.

**Figure 4:**
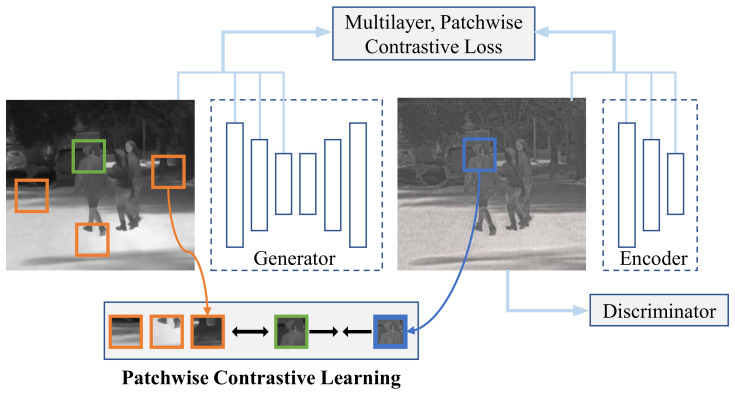
Illustration of the patchwise contrastive learning progress used in [56]. A generated intermediate image patch (marked with a blue box) is strongly associated with the corresponding patch (colored green) in the source image via contrastive learning, while disassociated from the other random patches. In this way, the generated intermediate domain is forced to preserve the source local content.

In particular, the CUT method is able to associate the corresponding local content during the training progress via patchwise contrastive learning, as shown in Figure 4. A patch is sampled from the generated intermediate image as “query” and compared to the “positive” source patch at the same location, while “negative” patches are randomly selected at different locations within the same image. Thus, contrastive learning aims to minimize the distance between the query and positive patches sharing the same content but maximize the distance otherwise. As shown in Figure 4, the generated image shows a different image style compared to the source image (e.g., with higher contrast and more textures) while keeping the consistent local content.

**Figure 5:**
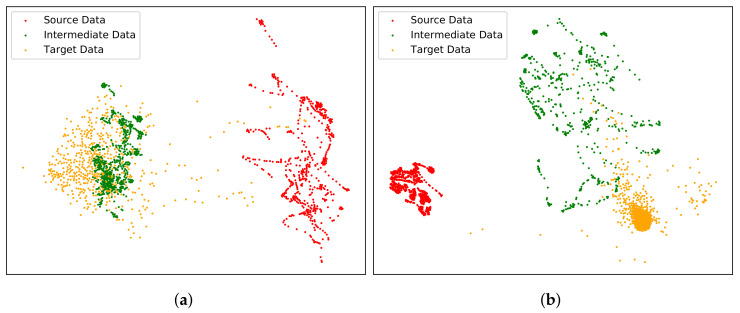
Visualization of the data distribution of source, intermediate and target domains. From every domain, 500 images are taken and their features represented as dots are extracted via t-SNE [57], where red, green and orange dots refer to the source, intermediate and target data respectively. (**a**) visual, (**b**) thermal.

The generated intermediate domain M is supposed to have a feature space distribution closer to the target T compared with the source domain S. Here, we present an example of the data distribution differences between the KAIST [9] and FLIR ADAS [12] datasets in Figure 5. The intermediate domains are generated using the above mentioned CUT method on visible and thermal modalities, respectively. The distribution of data is extracted and mapped to a two-dimensional feature space with the help of t-SNE [57]. As shown in Figure 5, the generated intermediate data are at a closer distance to the target data in feature space compared to the original data. It is worth noting that the clusters of source and target data in the thermal spectrum are denser than those in the visible spectrum. It is reasonable that the thermal images contains fewer detailed visual features than the visible images, leading to the denser clusters. The source thermal images in KAIST dataset have much fewer contrasts and texture features than the target FLIR ADAS dataset. Accordingly, the generated intermediate thermal data imitate the high-contrast and rich-texture source style, leading to more internal variations.

With the help of the intermediate domain, the whole adaption task S→T in the proposed UTL framework is divided into two phases, i.e., S→M and M→T, respectively. At the first phase of UTL, the intermediate images $\left\{X_{m}^{V};X_{m}^{T}\right\}$ share the same local image contents (e.g., pedestrian locations and sizes) with their corresponding source images $\left\{X_{s}^{V};X_{s}^{T}\right\}$, although the domain style (e.g., image contrast and texture) is transferred to match the target domain. Accordingly, by combining the generated intermediate images $\left\{X_{m}^{V};X_{m}^{T}\right\}$ and the manual training labels $Y_{s}$ from the source domain, we perform the supervised training of a detector as the initial adaptation process S→M. An initially adapted detector provides better detection results on the target images compared to the one trained only on source data. By evaluating the initially adapted detectors on the target data, we get the pseudo training labels for second-phase adaptation task M→T.

### 3.3. Illumination-Aware Label Fusion

Recently, pseudo labels have been widely used in image classification tasks to include the large amount of unlabeled data into the training process and improve the accuracy [58]. Normally, iterative fine-tuning is adopted to generate more pseudo training labels and help the convergence on the target domain, while the noisy labels do not significantly reduce the performance in the task of image classification. However, the whole task of pedestrian detection consists of not only the classification but also the localization subtask, which makes it quite sensitive to the inaccurate pseudo labels. Thus, how to select the most accurate pseudo labels is very important for the iterative fine-tuning of a pedestrian detector in our proposed UTL framework.

For the multispectral pedestrian detection task, the dual cameras capture aligned visible-thermal images, where different characteristic features are exhibited from different modalities. It is known that current multispectral pedestrian detectors extract fused features and can achieve better detection performance compared to any single-modality detectors. However, the generalization capability of a complex multispectral detector is worse than a single-modality detector. A multispectral detector is sensitive to both the visible and thermal domain gaps across different datasets. It is difficult to explicitly determine when and whether a multispectral detector works well. A visible detector generalizes well when it is evaluated in a new dataset consisting of only sunny daytime data. As for a thermal detector, it still sees pedestrians under low-illumination conditions. Thus, we adopt auxiliary pedestrian detectors which specialize in single modalities (visible and thermal respectively) to generate pseudo labels for the iterative fine-tuning of a multispectral pedestrian detector. Furthermore, the multispectral detector itself with updated parameters is also used to generate pseudo training labels for the next iteration.

When a pedestrian detector is applied to the target domain, an arbitrary detection result ${\hat{y}}^{i}$ is accompanied by a confidence score $c({\hat{y}}^{i})$ (*i = V, T* or *F*, representing the visible, thermal and multispectral modalities respectively). All the three sets of candidate pseudo labels are selected according to the confidence score:

(1) $$\left\{\begin{array}{c} Y^{V}=\left\{{\hat{y}}^{V}\in {\hat{Y}}^{V}:c\left({\hat{y}}^{V}\right)>c_{thr}\right\}\\ Y^{T}=\left\{{\hat{y}}^{T}\in {\hat{Y}}^{T}:c\left({\hat{y}}^{T}\right)>c_{thr}\right\}\\ Y^{F}=\left\{{\hat{y}}^{F}\in {\hat{Y}}^{F}:c\left({\hat{y}}^{F}\right)>c_{thr}\right\}, \end{array}\right.$$

where $c_{thr}$ is a confidence threshold. In this paper, the threshold $c_{thr}$ is empirically set to 0.9 and only detections with high confidence scores are chosen for the subsequent fusion.

Among all the pseudo labels generated by the mentioned detectors in an iteration, there are false labels as well as inaccurate detections. Usually, a visible detector is sensitive to the environmental illumination of the sensed image, resulting in false labels when there is insufficient lighting. In addition, a multispectral detector is supposed to have better performance over any auxiliary detectors when the illumination level is high because of the complimentary visual and thermal features, while the thermal detector generates slightly worse detection results than the visible one restricted by lack of appearance details. As for the case of low illumination level, the thermal detector achieves the best performance while the outputs of a visible detector are unreliable [18].

**Figure 6:**
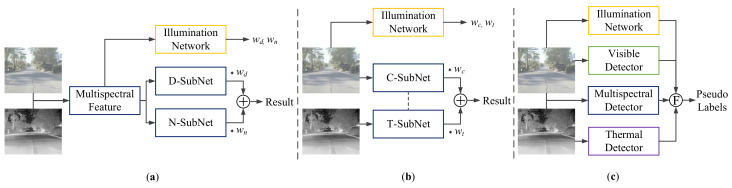
Illustration of the difference between existing illumination-aware weighting mechanisms and our illumination estimation network for pseudo label fusion. (**a**,**b**) are the illumination-aware weighting mechanisms proposed in [18,43] for supervised training respectively, where *w* represents the illumination weight and ⊕ indicates the operation of matrix addition. The final result contains both the confidence score and predicted bounding box, calculated as the weight sum of corresponding items from two modalities respectively. (**c**) illustrates our illumination-aware label fusion mechanism, where Ⓕ indicates the pseudo label fusion process.

Based on the above considerations, we propose an illumination-aware label fusion strategy to fuse the pseudo labels according to their priorities for the best quality. As shown in Figure 6, there are two types of illumination-aware networks [18,43] integrated into supervised multispectral pedestrian detection tasks. The detection results (both bounding boxes and confidence scores) are fused as the weight sum of two corresponding output from the subnetworks, as shown in Figure 6a,b.

Compared to the built-in network that is easily affected by the varying multispectral feature maps, an independent illumination network with fixed parameters is more robust for the unsupervised transfer learning task. In our UTL framework, candidate pseudo labels are generated from both single-modality and multispectral detectors, where there are inevitable false or inaccurate labels. The illumination-aware weighting mechanism brings accumulated errors and is not suitable for the UTL task. In this paper, the candidate pseudo labels get fused by keeping only the labels with the highest priorities determined by the estimated environmental level. To achieve this, we introduce the use of an independent illumination estimation network (IEN) to guide the pseudo label fusion in this paper.

In particular, the IEN is trained on the source domain and used directly in the target domain. The proposed IEN only takes visible images as inputs because the thermal camera is not sensitive to illumination. Considering that there is no ground-truth labels for illumination information in the datasets, we take the known binary classes daytime/nighttime as the training labels for IEN, i.e., a daytime image labeled as 1 and a nighttime one as 0. For an arbitrary visible image $x_{i}^{V}$, the output of IEN is an estimated environmental illumination level $\ell \left(x_{i}^{V}\right)\in \left[0,1\right]$, where $\ell (x_{i}^{V})>0.5$ means the estimated illumination level is high. The construction of IEN is as follows: A visible input image is resized to 32×32 and followed by two convolutional layers with 5×5 kernels, while a 2×2 max pooling layer follows each convolutional layer. Three subsequent fully-connected layers with 120, 84, and 2 neurons, respectively, are used to classify the input. Besides, a dropout layer with a probability of 0.5 is inserted after the first fully-connected layer. ReLu is also adopted to overcome the vanishing gradient problem. The network is trained with the binary cross-entropy loss, and the softmax function is used to generate the output estimated illumination level.

Here, we utilize IEN as an estimator of the real environmental illumination level to guide the following label fusion process. Priorities are assigned to the candidate pseudo labels from different modalities, according to the estimated illumination output of IEN. For instance, a detected pedestrian in the form of a bounding box from the visible detector may have the corresponding detection from the thermal detector, i.e., with an Intersection Over Union (IoU) of bounding boxes greater than 0.5, while the visible detection usually is more reliable for images with high illumination levels owing to the complete visual appearance details. Hence, we assign a higher priority to the visible pseudo label and abandon the corresponding low-priority thermal label. The overall priority order of the proposed illumination-aware label fusion strategy is assigned as follows:

(2) $$\left\{\begin{array}{cc} P\left({\hat{y}}^{F}\right)>P\left({\hat{y}}^{V}\right)>P\left({\hat{y}}^{T}\right), & \mathrm{when}\;\ell (x_{t}^{V})>0.5\\ P\left({\hat{y}}^{T}\right)>P\left({\hat{y}}^{F}\right), & \mathrm{when}\;\ell (x_{t}^{V})\leq 0.5, \end{array}\right.$$

where $P({\hat{y}}^{F})$, $P({\hat{y}}^{V})$ and $P({\hat{y}}^{T})$ represent the priority order of candidate pseudo labels from multispectral, visible and thermal detectors respectively, and $\ell (x_{t}^{V})$ is the output of the proposed illumination estimation network. For images estimated with a high illumination level, pseudo labels from the multispectral detector receive the highest priority, and the thermal detections are assigned the lowest priority. When the estimated illumination level $\ell (x_{t}^{V})\leq 0.5$, the visible detection will not be used. On top of the assigned priority of each candidate pseudo label, the proposed label fusion strategy select one bounding box with the highest priority from the overlapping bounding boxes of different modalities. The selected bounding box serves as a pseudo training annotation for fine-tuning of detectors to the target domain T.

To avoid the overfitting of iterative training, the early stopping mechanism is adopted. We evaluated different maximum iteration values and found that the detection performance reaches the best after three iterations. Thus, the number of maximum iterations is set to three empirically in this paper. At the end of the iterative adaptation process, a multispectral pedestrian detector has been fine-tuned to the target domain T.

## 4. Experiments and Results

We evaluate the proposed unsupervised transfer learning (UTL) framework on various visible-thermal multispectral datasets for the pedestrian detection task in this section. The datasets used as the source and target domains are introduced in Section 4.1. The related experimental setup, including the implementation details, is given in Section 4.2. The main experimental results with state-of-the-art comparisons are presented in Section 4.3. Moreover, ablation studies of the intermediate domain adaptation and illumination-aware label fusion strategy are conducted in Section 4.4.

### 4.1. Datasets and Metrics

**KAIST** [9] is one of the most widely used large-scale multispectral pedestrian detection datasets. The dataset is divided into daytime and nighttime scenarios by the original authors according to the recorded timestamps and provides visible-thermal image pairs that are aligned well with a resolution of 640×512. Using the same settings as in [18], a set of 7601 RGB-thermal image pairs is utilized as the training set, and 2252 images pairs are adopted as the test set. The involved images suffer from various serious challenges, such as illumination changes and occlusions. Specifically, KAIST is chosen as the source and the target dataset separately in the different groups of experiments in this paper. Since the original annotations of the test set have the problem as well as missing bounding boxes, we choose the widely used improved annotations provided by [10] for a fair comparison in our experiments.

**CVC-14** [38] is another widely used large-scale multispectral dataset containing visible-thermal image pairs, where the visible images are in the grayscale form instead of RGB. There is also the division of daytime and nighttime images. There are 7085 image pairs in the training set and 1433 frames in the test set, with a resolution of 640×480. However, a large portion of the image pairs face the problem of misalignment. The primary goal of this paper is to design an unsupervised transfer learning framework instead of a specific multispectral detector to handle the misalignment challenge. To perform a fair and clear comparison, CVC-14 is only used as the source dataset in our experiments. Accordingly, an auxiliary single-modality pedestrian detector is trained with its corresponding modality-specific annotations, while the training labels for the thermal images are used as the ground truth for the training of a multispectral detector. Especially, the multispectral detector is not adopted to generate pseudo labels in the first iteration of our proposed iterative fine-tuning phase for the sake of quality assurance of generated pseudo labels.

**FLIR ADAS** [59] is a recently published multispectral object detection dataset. The RGB-thermal image pairs are collected in the Santa Barbara, CA, USA, with a resolution of 640×512. The dataset includes images captured under different lighting conditions, while the labels of daytime/nighttime are not directly given. The FLIR ADAS dataset contains not only pedestrian annotations but also annotations for other detected objects. In this paper, we only use the labeled pedestrians. Since there are some misalignment problems between the visible and thermal images among the original dataset, a sanitized version [12] of the dataset in which misaligned image pairs were manually removed, is adopted in our experiments. The sanitized dataset contains 4129 well-aligned image pairs for training and 1013 image pairs for testing. We only treat FLIR ADAS as the target dataset, due to its relatively small amount of training images and pedestrians involved.

**Evaluation Metric**. Following the guidelines by the authors of [9], the reasonable setting is used for the evaluation, including all non-occluded pedestrians larger than 55 pixels. A detected pedestrian is considered as a correct match (true positive) if the Intersection over Union (IoU) of the detection and corresponding ground-truth bounding box is greater than 0.5, which is the common choice of IoU threshold for pedestrian detection. Log-Average Miss Rate (LAMR) is utilized as the detection performance metric for consistency with the literature [9,10,15]. The LAMR metric computes the averaging miss rate of pedestrians against 9 evenly distributed false positives per image (FPPI) over the log range of $\left[10^{-2},10^{0}\right]$. A lower value of LAMR means a better detection performance.

### 4.2. Experimental Setup

**Multispectral pedestrian detector.** All the detectors used in this paper are implemented based on Faster R-CNN [25]. We utilize the Feature-Map Fusion method [17] concatenating feature maps of the thermal and visible branches in Faster R-CNN with a backbone of VGG16 [60] to form a multispectral pedestrian detector, which follows the successful design of Halfway Fusion [10]. After the concatenation operation, a convolutional layer called Network-in-Network (NIN) with 1×1 kernel is attached to reduce the dimension as well as to fit into the standard Faster R-CNN architecture. It is efficient to fuse individual features maps of the two modalities to generate slightly high-level multispectral features. All the demonstrated experiments related to any stage of our UTL framework adopt the multispectral pedestrian detector based on Feature-Map Fusion.

**Training details.** The backbone VGG16 of Faster R-CNN is pre-trained on ImageNet dataset [61]. At each training stage, horizontal flipping is adopted as a basic data augmentation operation, and the parameter update of detectors is performed with the help of stochastic gradient descent (SGD). At the stage of initial adaptation, the training progress contains 6 epochs with a learning rate (LR) of 0.001 for the first four epochs and LR 0.0001 for the last two epochs. As for the iterative fine-tuning stage, we fine-tune a detector for the first epoch with LR 0.001 and one more epoch with LR 0.0001 at each iteration. The above mentioned training settings apply to both multispectral and auxiliary pedestrian detectors in all the experiments. We follow the default settings [56] to train the CUT model for intermediate domain adaptation, where the unpaired input instances are constructed with visible images from the source and target domains separately. All the input images are resized to 480×480 and then cropped to 256×256 to fit into the CUT model and keep the image details. As for the inference stage, the images are taken as their original resolutions to generate the intermediate images with the same resolution as the source images. In this way, the training labels from the source domain can be used directly to supervise the initial training based on the generated intermediate images. Furthermore, the proposed illumination estimation network is trained with Adam optimizer for two epochs with LR 0.0001, using only visible images in the source domains. For the unsupervised transfer learning experiments, the ground-truth annotations of the target training set are abandoned to form a target domain without any manual training labels.

### 4.3. Main Results

Here, we provide the experimental results for multispectral pedestrian detection using the proposed UTL framework and compare the performance with state-of-the-art methods. As stated in Section 4.1, the CVC-14 and KAIST datasets are adopted as source datasets, while the KAIST and FLIR ADAS datasets are set as the target. Given that there is limited UTL research in the multispectral pedestrian detection area, we report the results of the only available state-of-the-art method U-TS-RPN [15] on KAIST. What is more, we compare the same detector’s performances trained in three different ways: fully supervised trained on the target domain, trained on the source domain and unsupervised transferred to the target domain using our proposed UTL method, to see the gain on both datasets.

**Figure 7:**
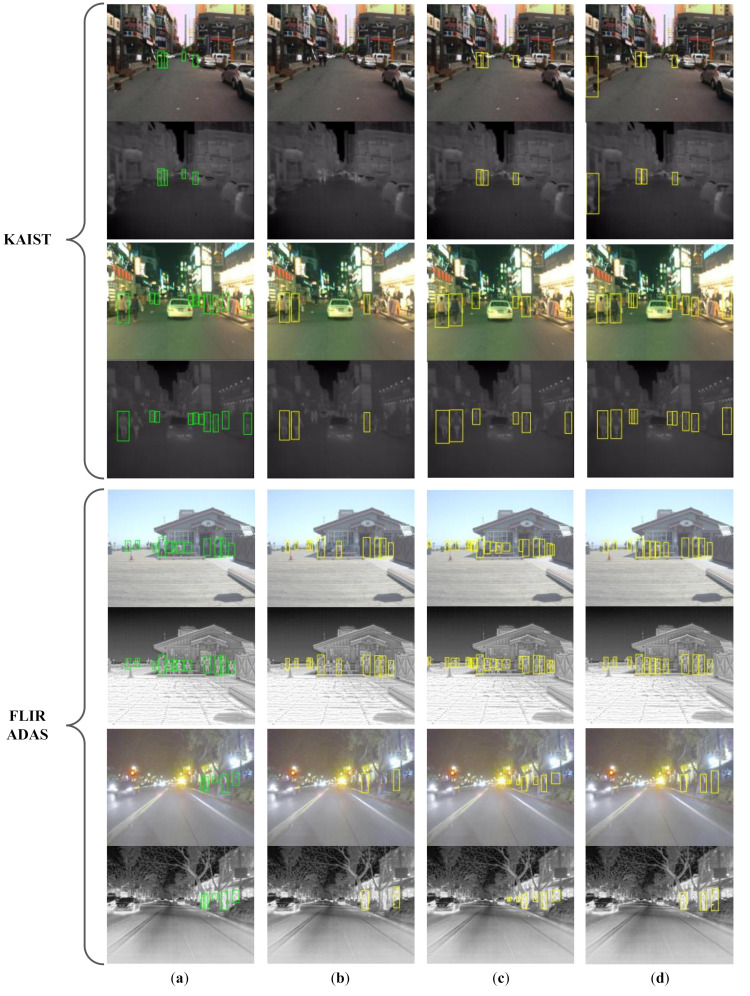
Examples of pedestrian detection results. (**a**) The ground truth. Detection results of the detector (**b**) trained only on CVC-14, (**c**) trained on the target dataset with manual annotations and (**d**) adapted from CVC-14 to the target dataset using our framework. The shown detections are test results from KAIST and FLIR ADAS datasets containing various illumination conditions.

The experimental results on KAIST and FLIR ADAS datasets are presented in Table 1 and Table 2, respectively. Examples of pedestrian detection results are shown in Figure 7.

In our experiments, a method classified as “supervised training” category trains a pedestrian detector on the target images using the corresponding ground-truth training annotations. Specifically, the classical Halfway Fusion method is re-implemented in this paper according to the original settings [10]. From Table 1 we can see that the performances of two deep learning-based detectors surpass the classical handcrafted multispectral detector ACF + T + THOG [9]. What is more, as shown in both Table 1 and Table 2, the Feature-Map Fusion detector outperforms the Halfway Fusion detector on both KAIST and FLIR ADAS datasets, owing to the high-level feature concatenation from individual feature maps. As a result, we adopt Feature-Map Fusion as the baseline multispectral detector in all the following experiments.

For results reported in the category of “unsupervised transfer learning”, the whole training progress of the pedestrian detector does not involve any manual labels from the target dataset, and its cross-dataset detection performance is demonstrated. Besides, the results of the same detector trained with only source data and tested on the target test set, are marked as “source only”. An unsupervised adapted detector is supposed to have a significantly better cross-dataset performance (i.e., lower LAMR), compared to the detector trained on the source dataset only.

On the KAIST dataset, we use *CVC-14 → KAIST* as the domain pair to test our proposed UTL framework. For comparison experiments, the results of state-of-the-art unsupervised multispectral feature learning method U-TS-RPN [15] is reported in Table 1. In particular, the pseudo training labels for U-TS-RPN are provided by the original authors, and the results reported are fine-tuned on the KAIST training set, based on the same multispectral pedestrian detector as used in our framework for a fair comparison. The proposed method introduces a clearly lower LAMR (19.98% vs. 30.07%) for all-day scenes. Compared to the performance of detector trained on source data only, our UTL framework significantly reduces the LAMR from 51.94% to 19.98% for all-day scenarios. Furthermore, our method achieves a similar detection performance compared to fully supervised training, without using any training annotations from the target domain, making the deployment scalable and avoiding the human re-labeling work. Specially, our performance on nighttime images is significantly superior (15.78% vs. 26.17%), while our method reports a performance that is slightly worse in the daytime scenario.

To evaluate the effectiveness on FLIR ADAS dataset, two pairs of cross-dataset experimental are conducted, i.e., *CVC-14 → FLIR ADAS* and *KAIST → FLIR ADAS*, and the results are presented in Table 2. Since FLIR ADAS dataset is relatively new and there is no unsupervised related work tested on it, we validate the effectiveness of the proposed framework, by comparing with detectors fully trained on the target data with manual annotations as well as those trained on source data only without adaptation. As we can see, our adapted detectors surpasses the unadapted detectors (trained on source only) by a large margin. Notably, the proposed method reports a comparable detection performance with the supervised trained result (LAMR 33.16% vs. 31.41%) when the source dataset is CVC-14, and an apparently worse result for the case of KAIST (LAMR 44.19%) because there is a large domain gap between the KAIST and FLIR ADAS datasets. We will analyze the domain gap problem in Section 4.4.1.

Overall, the results presented in this section reflect that the proposed framework is robust enough to perform the unsupervised adaptation across different multispectral pedestrian detection datasets. Compared with U-TS-RPN [15], our framework achieves significantly better detection performance with the help of intermediate domain adaptation to tackle the domain shift problem. The two-stage adaptation strategy makes the proposed framework capable of reporting similar results with supervised training, while reducing the requirements of labeling new data for target domain and effectively increasing its portability.

### 4.4. Ablation Study

Here, two essential ablation studies are conducted to examine the effects of the intermediate domain adaptation and illumination-aware label fusion. In order to show each effect of the two key steps clearly, we report the experimental results solely using the related step, respectively.

**Figure 8:**
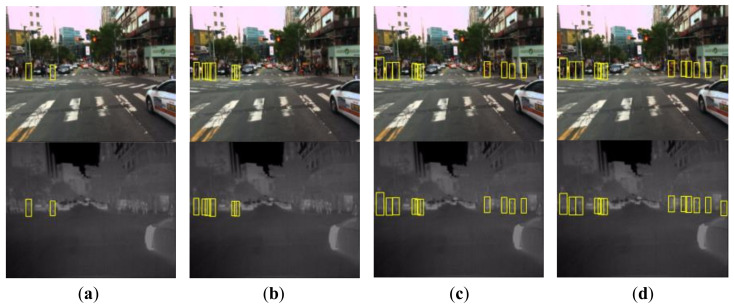
Qualitative examples for the ablation study. Detection results of the detector (**a**) trained only on CVC-14 dataset, (**b**) adapted initially on the generated intermediate images, (**c**) fine-tuned iteratively from CVC-14 to KAIST dataset without using intermediate images and (**d**) adapted from CVC-14 to KAIST dataset using our whole framework.

Figure 8 shows a qualitative result for our ablation study. Comparing Figure 8b,c with Figure 8a, respectively, we can clearly see that each of the two steps improves the detection performance. The whole framework benefits from both two steps and achieves the best performance, as shown in Figure 8d.

#### 4.4.1. Effects of Intermediate Domain Adaptation

Usually, the data distribution between different datasets varies greatly, making a bad direct evaluation performance across datasets. In this paper, the use of intermediate images generated from the source dataset forms an intermediate domain bridging the gap between the source and target domains. The intermediate domain consists of both the original source training annotations and the generated intermediate images with a domain style similar to the target domain. We take advantage of both the source and target domain knowledge via training a pedestrian detector on the intermediate domain.

To validate the effectiveness of intermediate domain used in the initial adaptation phase, we conduct three groups of analytic experiments, i.e., *CVC-14 → KAIST*, *CVC-14 → FLIR ADAS* and *KAIST → FLIR ADAS*. Specifically, the initially adapted multispectral pedestrian detector, which is trained on the generated images of the intermediate domain, is applied to the test set of the corresponding target dataset and marked as “Initial Adaptation (w/ID)” in Table 3. Moreover, histogram matching is used as a weak version of the intermediate domain adaptation technique. In our experiments, image-to-image histogram matching is conducted according to the same source-to-target correspondence as the CUT model’s inference stage. The reported results with the help of histogram matched synthetic images are listed as “Initial Adaptation (w/HM)” and the performances of detector trained on source data only are also reported in Table 3.

From all the three groups of experiments in Table 3, we find that the idea of initial adaptation based on synthetic (both histogram matched and intermediate) images boosts the cross-domain detection performance. The reason could be that a modern detector using DCNN-based architecture tends to learn domain-specific knowledge to reach the best performance on a specific domain. However, this kind of domain-specific knowledge reduces the generalization ability. The synthetic images, which have a similar image style to the target images, provide the target domain knowledge during the initial adaptation step in our UTL framework. As a traditional way of adjusting the similarity of intensity distribution, histogram matching focuses on pixel-level processing and could not learn the global environmental knowledge, making it not as good as the proposed intermediate domain adaptation method. It is worth noting that initial adaptation from KAIST to FLIR ADAS helps LAMR change from 64.64% to 52.41%, which is a much larger margin compared to the one adapted from CVC-14 with LAMR reducing from 43.92% to 39.16%. Thus, even though the source data might have a tremendous domain gap to the target data, the intermediate domain adaptation can effectively reduce the gap and boost the detection performance.

#### 4.4.2. Effects of Illumination-Aware Label Fusion

Pseudo labels provide functionality for training/fine-tuning a detector on the target domain without any manual training labels. In practice, the visible branch in a multispectral setup is sensitive to the illumination conditions caused by the diurnal cycle, shadow, and extreme weather, leading to false or inaccurate pseudo labels. Our proposed illumination-aware label fusion strategy relies on the fact that the most accurate pseudo labels come from different modalities according to the illumination conditions.

To investigate the influence of the strategy mentioned above, we hereby report the detection performance using label fusion based on estimated illumination against the daytime/nighttime information in Table 4. Since there is no division of daytime/nighttime in FLIR ADAS dataset, only the quantitative experiment *CVC-14 → KAIST* is conducted in this paper. When there is no illumination knowledge available, we let the multispectral detector generate pseudo labels to fine-tune itself without using auxiliary detectors, which is the common form of iterative self-training to adapt to the target domain. The use of daytime/nighttime as illumination information, makes the reported LAMR of all-day scenarios decrease from 34.50% to 23.09%, which confirms the effectiveness of fusing auxiliary pseudo labels. Furthermore, the images are assigned with different illumination levels from the IEN output in the UTL framework, according to their real environment lighting conditions rather than the division by time. The overall detection performance of a detector iteratively fine-tuned with the illumination-aware fused pseudo labels is 2% better than daytime/nighttime-aware fusion.

## 5. Conclusions

In this paper, we propose a novel unsupervised transfer learning framework for visible-thermal multispectral pedestrian detection. Our goal was to develop a general framework to adapt a multispectral pedestrian detector to the target dataset without using any manual target annotations. The main novelty of this paper is the two-step adaptation solution. The initial adaptation is performed by training the detector on an intermediate domain, leveraging both the source and target domain knowledge to reduce the domain gap. After that, an iterative process is conducted to fine-tune the detector based on fused pseudo labels from different modalities, according to the proposed illumination-aware fusion strategy. The extensive experimental results demonstrate the effectiveness of our framework on both KAIST and FLIR ADAS datasets. As can be seen by the results presented, our method allows an effective adaptation to new environments or datasets without the necessity of manually labeling a new training set, making the multispectral detector flexible and generalizable. This provides the possibility of using the proposed framework under a high variety of scenarios without needing the inputs of specialists.

Opportunities for future work lie in the direction of investigating pedestrian-centric intermediate domain representation and feature alignment. We will also explore the potential direction of extending the framework into relevant object detection tasks in traffic scenarios such as vehicle detection to support the smart city development.

## Figures and Tables

**Table 1:** Detection performance comparisons on KAIST dataset. The original manual annotations are used to perform the supervised training. For the results of U-TS-RPN [15], the pseudo training labels are provided by the original authors and detections are generated by the fine-tuned detector accordingly.

Methods	LAMR (Lower, Better)		
	All	Daytime	Nighttime
*Supervised training*			
ACF + T + THOG [9]	47.25%	42.44%	56.17%
Halfway Fusion [10]	26.14%	24.08%	29.01%
Feature-Map Fusion	21.27%	18.63%	26.17%
*Source only:*			
Feature-Map Fusion (*CVC-14*)	51.94%	53.83%	44.76%
*Unsupervised transfer learning:*			
U-TS-RPN [15]	30.07%	31.59%	26.78%
Ours (*CVC-14 → KAIST*)	19.98%	22.17%	15.78%

**Table 2:** Detection performance comparisons on FLIR ADAS dataset. The supervised training is performed on FLIR ADAS dataset directly using manual labels of the “person” class.

Methods	LAMR (Lower, Better)
*Supervised training*	
Halfway Fusion [10]	40.43%
Feature-Map Fusion	31.41%
*Source only:*	
Feature-Map Fusion (*KAIST*)	64.64%
Feature-Map Fusion (*CVC-14*)	43.92%
*Unsupervised transfer learning:*	
Ours (*KAIST → FLIR ADAS*)	44.19%
Ours (*CVC-14 → FLIR ADAS*)	33.16%

**Table 3:** Ablation study of intermediate domain (ID) in terms of Log-Average Miss Rate (LAMR). Results of detectors trained with original source images and histogram matched (HM) synthetic images are reported for comparisons. The best results are highlighted in bold.

Source → Target	Methods	LAMR (Lower, Better)		
		All	Daytime	Nighttime
*CVC-14 → KAIST*	Detector Trained on CVC-14	51.94%	53.83%	44.76%
	Initial Adaptation (w/HM)	43.28%	46.83%	33.79%
	Initial Adaptation (w/ID)	**41.07%**	**45.11%**	**29.20%**
*KAIST → FLIR ADAS*	Detector Trained on KAIST	64.64%	-	-
	Initial Adaptation (w/HM)	60.13%	-	-
	Initial Adaptation (w/ID)	**52.41%**	-	-
*CVC-14 → FLIR ADAS*	Detector Trained on CVC-14	43.92%	-	-
	Initial Adaptation (w/HM)	40.51%	-	-
	Initial Adaptation (w/ID)	**39.16%**	-	-

**Table 4:** Ablation study of illumination-aware label fusion. The results using known daytime/nighttime information to determine the fusion strategy are listed for comparison. When there is no illumination information available, the multispectral pedestrian detector generates pseudo labels and adapts itself accordingly. The best results are highlighted in bold.

Methods	LAMR (Lower, Better)		
	All	Daytime	Nighttime
Without illumination info	34.50%	39.21%	24.49%
With daytime/nighttime info	23.09%	24.55%	17.74%
With estimated illumination	**21.09**%	**23.32**%	**17.14**%

## Data Availability

Not applicable.
